# A Co-Expression Network Reveals the Potential Regulatory Mechanism of lncRNAs in Relapsed Hepatocellular Carcinoma

**DOI:** 10.3389/fonc.2021.745166

**Published:** 2021-08-31

**Authors:** Yuan Fang, Yang Yang, XiaoLi Zhang, Na Li, Bo Yuan, Li Jin, Sheng Bao, MengGe Li, Dan Zhao, LingRui Li, Zhong Zeng, HanFei Huang

**Affiliations:** ^1^Organ Transplantation Center, The First Affiliated Hospital of Kunming Medical University, Kunming, China; ^2^Department of Otorhinolaryngology, The First Affiliated Hospital of Kunming Medical University, Kunming, China; ^3^Gastrointestinal and Hernia Surgery, First Affiliated Hospital of Kunming Medical University, Kunming, China; ^4^Department of Medical Oncology, The First Affiliated Hospital of University of Science and Technology of China (USTC), Division of Life Sciences and Medicine, University of Science and Technology of China, Hefei, China; ^5^Laboratory of Oncogenes and Related Genes, Shanghai Cancer Institute, Renji Hospital, Shanghai Jiao Tong University School of Medicine, Shanghai, China; ^6^School of Automation Science and Engineering, South China University of Technology, Guangzhou, China

**Keywords:** hepatocellular carcinoma, lncRNA, WGCNA, prognosis, lncRNA-mRNA network, relapse

## Abstract

**Background:**

The mechanistic basis for relapsed hepatocellular carcinoma (HCC) remains poorly understood. Recent research has highlighted the important roles of long non-coding RNAs (lncRNAs) in HCC. However, there are only a few studies on the association between lncRNAs and HCC relapse.

**Methods:**

Differentially expressed lncRNAs and mRNAs between a primary HCC group and relapsed HCC group were identified using the edge R package to analyze the GSE101432 dataset. The differentially expressed lncRNAs and mRNAs were used to construct a lncRNA–mRNA co-expression network. Weighted gene co-expression network analysis followed by Gene Ontology (GO) enrichment analyses were conducted on the database. Furthermore, correlation and survival analyses were performed using The Cancer Genome Atlas database, and expression in the clinical samples was verified by qRT-PCR. Thereafter, we inputted the genes from the two groups into the HCC TNM stage and tumor grade database from TCGA. Finally, we performed Kaplan–Meier survival analysis on the lncRNAs related to relapsed HCC.

**Results:**

In this study, lncRNAs and mRNAs associated with HCC relapse were identified. Two gene modules were found to be closely linked to this. The GO terms in the yellow and black modules were related to cell proliferation, differentiation, and survival, as well as some transcription-related biological processes. Through qRT-PCR, we found that the expression levels of LINC00941 and LINC00668 in relapsed HCC were higher than those in primary HCC. Further, mRNA levels of *LOX*, *OTX1*, *MICB*, *NDUFA4L2*, *BAIAP2L2*, and *KCTD17* were changed in relapsed HCC compared to levels in primary HCC. In addition, we verified that these genes could predict the overall survival and recurrence-free survival of HCC. Moreover, we found that LINC00668 and LINC00941 could affect tumor grade and TNM stages. In total, we identified and validated two lncRNAs (LINC00941 and LINC00668) and six mRNAs (*LOX*, *MICB*, *OTX1*, *BAIAP2L2*, *KCTD17*, *NDUFA4L2*) associated with HCC relapse.

**Conclusion:**

In summary, we identified the key gene modules and central genes associated with relapsed HCC and constructed lncRNA–mRNA networks related to this. These genes are likely to have potential prognostic value for relapsed HCC and might shed new light on novel biomarkers or diagnostic targets for relapsed HCC.

## Introduction

Hepatocellular carcinoma (HCC) is the sixth most prevalent cancer in the world (1). Furthermore, the incidence rate of HCC showed its greatest increase from 2007 to 2016 at 2% to 3% per year, although this rate has decreased as compared to that in previous years. For all stages combined, the 5-year relative survival rate is the lowest (18%) for HCC (2). Although patients receive curative treatment for early HCC, up to 70% of them might experience relapse in the liver after 5 years (3). Therefore, we aimed to explore the mechanism of HCC relapse and search for prognosis-associated long non-coding RNAs (lncRNAs), which are of great significance as they provide useful information for the diagnosis and prognosis of HCC.

lncRNAs are a group of non-coding RNAs that are more than 200 nucleotides in length and have no ability to encode proteins. An expanding body of evidence shows that lncRNAs modulate gene expression at multiple levels through many complex mechanisms. In addition to their well-established role as regulators of transcription, lncRNAs are also effective regulators of pre-mRNA splicing, mRNA decay, and translation (4). Portions of lncRNAs are specifically expressed in different tissues and cancers (5). In addition, lncRNAs are involved in the pathological process of tumors and act *via* mechanisms, such as cis-regulation at enhancers, chromatin reprogramming, and the post-transcriptional regulation of mRNA processing (6).

The emergence of wide-ranging cancer projects, such as The Cancer Genome Atlas (TCGA) research network project and Gene Expression Omnibus (GEO) has provided an excellent opportunity to characterize lncRNAs in various cancers. Furthermore, bioinformatics analysis has been used to establish lncRNA features with prognostic value, such as the identification of three lncRNA prognostic markers in ovarian cancer according to genome-wide copy number variation (7). Additionally, a few survival-related lncRNA signatures have been discovered in malignant tumors of the digestive system, such as gastric cancer (GC) (8, 9) and pancreatic cancer (PC) (10). Lai et al. reported that AFDN-DT, a lncRNA that is repressed in GC, functions as a tumor suppressor by inhibiting cell growth and metastasis through the transcriptional repression of genes involved in metastasis. At the same time, they demonstrated the tumor suppressive role of AFDN-DT in GC and elucidated the transcriptional regulatory role of tumor suppressive lncRNAs, which can serve as potential prognostic markers for GC (9). In a study by Han et al., LINC00514 silencing inhibited PC cell proliferation, migration and invasion, whereas its overexpression promoted these processes. Moreover, LINC00514 knockdown remarkably inhibited PC development and metastasis *in vivo*. In addition, increased LINC00514 expression was significantly associated with the clinical progression and prognosis of PC patients (11). At present, whereas a few reports have focused on the bioinformatics analysis of HCC (12–14), few are related to HCC relapse. In a study by Minzhen et al., functional assays revealed that lncRNA PRR34-AS1 promotes HCC cell proliferation, migration, invasion, and epithelial–mesenchymal (EMT) transition processes *in vitro* and facilitates tumor growth *in vivo* (15). Despite these studies, the functions of lncRNAs in relapsed HCC remain unclear. Therefore, a comprehensive analysis of HCC relapse-associated lncRNAs is necessary to reveal possible biomarkers and/or potential prognostic targets.

In this study, we repurposed and integrated HCC data from GSE101432 to identify differentially expressed lncRNAs. Thereafter, we profiled the co-expression network, and the expression data were analyzed through a weighted gene co-expression network analysis (WGCNA) to identify hub lncRNAs as potential prognostic biomarker candidates. Finally, relevant lncRNAs were then validated through correlation analysis between lncRNAs and co-expressed mRNA, qRT-PCR in a cohort of patients, and Kaplan–Meier analysis to verify the potential predictive role of lncRNA candidates. The in-depth analysis conducted in this study might provide novel insights into the relapse for HCC, along with new biomarkers and prognostic targets for this disease. However, further studies are needed to determine the potential use of these genes as biomarkers for this purpose.

## Materials and Methods

### Data Downloading and Preprocessing

The expression profile of genes associated with HCC (GSE101432) was obtained from the NCBI GEO database (https://www.ncbi.nlm.nih.gov/geo/). Furthermore, the GEPIA 2 database (http://gepia2.cancer-pku.cn/) was used to analyze the gene expression profiles from TCGA dataset (https://tcga-data.nci.nih.gov/tcga/) and the Genotype-Tissue Expression (https://gtexportal.org/) projects (16). In addition, since the data were downloaded from the GEO public database, no further approval from the ethics committee was required. Data processing was carried out in accordance with the TCGA Human Subject Protection and Data Access Policy.

### Read Alignments and Analysis of Differentially Expressed Genes (DEGs)

The clean reads were aligned with the human GRch38 genome using tophat2, allowing up to four mismatches (17). Uniquely mapped reads were ultimately used to calculate the read number and reads per kilobase million (RPKM) for each gene. The gene expression levels were evaluated based on the RPKM. The software edgeR (18), which is specifically applied to analyze the differential expression of genes, was used to screen the RNA-seq data for DEGs. A fold-change ≥2 or ≤0.5 and a false discovery rate (FDR) ≤0.05 were used as cutoffs.

### LncRNA Prediction and Direction Identification

To identify lncRNAs associated with relapsed HCC, we executed the pipeline described by Liu et al. (19), which was based on the cufflinks software developed by Trapnell et al. (20)] The pipeline and cufflinks are free, open-source software tools for gene research and comprehensive expression analysis of high-throughput RNA-seq data. Cufflinks (http://cufflinks.cbcb.umd.edu/) uses this genome map to assemble the reads into transcripts. Cuffdiff is part of the Cufflinks package. It takes aligned reads from two or more conditions and uses rigorous statistical analysis to report differentially expressed genes and transcripts. We calculated the coding potential score to filter the potential coding transcripts. When filtering single exon lncRNA, we set two thresholds, 1000 nt to obtain longer single exon lncRNAs and 500 nt to find more single-exon lncRNAs.

Then, we used the polyadenylation signal to detect the direction of lncRNA transcription. To begin, we selected the tail, which has more than 10 As or Ts, allowing for a 0.1 error rate. Then, we aligned reads longer than 20 nt with the genome sequence. If their poly(A) tail was derived from the genomic sequence (internal polyA), then the aligned reads were discarded. If it was located within 20 bp of the genomic locus, the end-aligned positions of the reads were clustered together, and the cluster was considered a polyadenylation site (PAS). Thereafter, we compared the predicted lncRNAs and the passed genome location. If the PAS was located downstream of the lncRNA, the direction of the lncRNA was the same as the reference direction, and vice versa. If there were poly (T) sites, the direction of lncRNA was reversed.

### WGCNA and Co-Expression Analysis

The lncRNAs and mRNAs that were differentially expressed among primary tumors, relapsed tumors, and benign adjacent tissue in the dataset were utilized for WGCNA (21) to reveal gene expression patterns. All expressed genes were considered input data. The eigengenes of each clustering module were utilized as the representative expression pattern of genes in each module. To investigate the regulatory relationship between lncRNAs and their target mRNAs, we calculated Pearson’s correlation coefficients. The adjacency matrix was then converted to a topological overlap matrix to minimize the effects of noise and false associations.

### Functional Enrichment Analysis

Gene Ontology (GO) terms were analyzed to determine the functional categories associated with the identified DEGs using the KOBAS 2.0 server (22). There are three main subtypes of GO analysis, cell composition, biological pathway, and molecular function. The Benjamini-Hochberg FDR controlling procedure and hypergeometric tests were used to define the enrichment of each term. Reactome pathway analysis was also conducted for functional enrichment analysis of the selected genes sets.

### Sample Collection

To further verify our results, samples of tumors and matched adjacent tissue were obtained from five patients with primary HCC and three patients with relapsed HCC who underwent tumor resection at the First Affiliated Hospital of Kunming Medical University from July 15, 2019, to December 20, 2020. This study was approved by the Ethics Committee of the First Affiliated Hospital of Kunming Medical University. We confirmed that all studies were conducted in accordance with relevant guidelines/regulations. A signed informed consent form was obtained from each patient or their family members, who were provided with a detailed explanation of the study. **Table 1** lists the clinical samples relevant information.

**Table 1:** Clinical sample information.

Clinical information	Sample
**Primary Tumor**	5
**Relapse Tumor**	3
**Age, median,** (mean)	54
**Gender, male**	4 (50%)
**Length of hospital stay** (days) (mean)	10-14 (12.875)
**Liver cirrhosis**	4 (50%)
**Classification of liver cancer**	
Hepatocellular carcinoma	8 (100%)
Intrahepatic cholangiocarcinoma	0
**Tumor diameter**	
<2cm	1 (12.5%)
2cm-5cm	5 (62.5%)
>5cm	2 (25.0%)
**HBV**	
Positive	4 (50%)
Negative	4 (50%)
**AFP**	
<400	4 (50%)
>400	4 (50%)
**Tumor location**	
Left liver	5 (62.5%)
Right liver	3 (37.5%)
**Tumor recurrence time** (months)	18,27,76
**Outcome**	
Cure	1 (12.5%)
Improve	6 (75%)
Death	1 (12.5%)

### qRT-PCR

The total RNA samples were isolated using TRIzol reagent (Invitrogen). Next, quantitative real-time reverse transcription-polymerase chain reaction (qRT-PCR) analysis was performed using SYBR Green Master Mix (ROX, Roche) and an ABI Quant Studio 5 system, and changes in gene expression were calculated using the comparative CT approach. Primers used in the study are listed in **Table 2**.

**Table 2:** Primers of lncRNA and mRNA for qRT-PCR.

Primers for Validated Genes			
Gene	lncRNA ID/GENE ID	Prime sequence (5’-3’)	
		Forward	Reverse
**RP11-334A14.8**	NONHSAT003288.2	AGGGAACCCCATTCTTCCAA	AGAGGTCTCAAGGCACAGAT
**LINC00668**	NONHSAT057143.2	GGGTCCAAGGGATCTGCAAG	CCGCCCAAGATTCCTCTAGC
**CTD-2369P2.5**	NONHSAT061043.2	GCACTTTCCCAAGGGTACAA	CCCTTGACCCTAACCTCTGA
**LINC00941**	NONHSAT027514.2	CATGTACGTTGCTATCCAGGC	CTCCTTAATGTCACGCACGAT
**AF124730.4**	NONHSAT081627.2	GCCACACAAATGTTCACCAC	GAGCAGAAGGACTCAGTGTG
**RP13-143G15.4**	NONHSAT115109.2	ACCCTTGCAACCTTTCTCAG	TGGCCACCTTGTAGGTTAGA
**RP11-973F15.2**	NONHSAT128496.2	GACAGCCCCCAAAGAACC	ATCCCCGGACTGCTGC
**RP5-967N21.11**	NONHSAT188398.1	TGGTATGTTTGTGTTCCATTTGC	AATGCGAGCACAAGATAATTTTT
**RASAL1**	8437	CAGCTCCCTGAATGTTCGC	TCCTCATCCAGCACGTAGAAG
**NDUFA4L2**	56901	CCTGAGCCCCAATGACCAATA	TCTGGCCGGTCCTTCTTCA
**KCTD17**	79734	AGTCGGACCGGGATGAGAC	CCATGCCGGAGGAAGTTCA
**BAIAP2L2**	80115	GCCGAGGTCTACTTCAGTGC	GCTGGGTGTCAGACATCTGC
**MDFI**	4188	CCACCGGAAGTTGCAGACA	GGAACTCGCAGAACAGGCA
**SLC44A5**	204962	GACTTTGGTGATCCAAGGACAT	GTCTGTAGGATAGGCTGCTCT
**GPR160**	26996	CCAGCCATCTACCAAAGCCTG	GCCAGTAACTCTGAATGCTGACA
**LOX**	4015	GCCGACCAAGATATTCCTGGG	GCAGGTCATAGTGGCTAAACTC
**NXPH4**	11247	AAGGTCTTCGGACGGCCTA	GCAGCGAAAACTTGAGGGTAT
**OTX1**	5013	GCGTCGTCGCTGAGTACAC	ACATGGGATAAGAGGCTGCTG
**GMNN**	51053	GCCCTGGGGTTATTGTCCC	AGCGCCTTTCTCCGTTTTTCT
**MICB**	4277	TCTTCGTTACAACCTCATGGTG	TCCCAGGTCTTAGCTCCCAG

### Statistical Analysis

Principal component analysis (PCA) analysis was employed by with the R software package factoextra (https://cloud.r-project.org/package=factoextra) for effective dimension reduction, pattern recognition, and exploratory visualization of high-dimensional data of the whole genome. After normalizing the reads by TPM (Tags Per Million) of each gene in samples, based on TPM (tags per million), an in house-script (sogen) was utilized for the visualization of next-generation sequence (NGS) data and genomic annotations. The pheatmap package (https://cran.r-project.org/web/packages/pheatmap/index.html) in R was used to carry out the clustering based on Euclidean distance. A Student’s t-test was used by in SPSS22.0 for comparisons between two groups. For results, a *P* value < 0.05 and FDR < 0.05 were saw asconsidered statistically significant. (*P < 0.05, **P < 0.01, ***P < 0.001, ****P < 0.0001)

Kaplan–Meier Plotter was used to analyze overall survival (OS) and recurrence-free survival (RFS). Hazard ratios (HR) and 95% confidence intervals were derived from a Cox proportional hazard model with stratified log-rank test.

## Results

### A Comprehensive Catalog of lncRNA and mRNA Genes in Primary and Relapsed Tumors and Benign Adjacent Tissue

**Figure 1:**
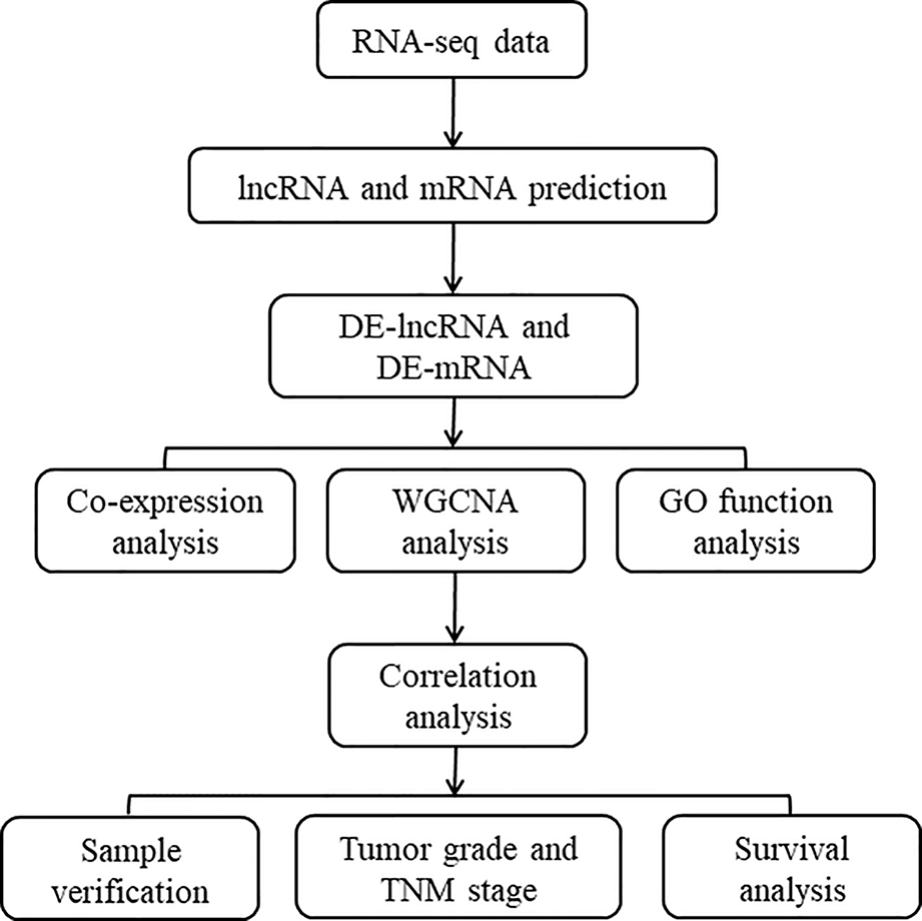
Flowchart of the study.

**Figure 2:**
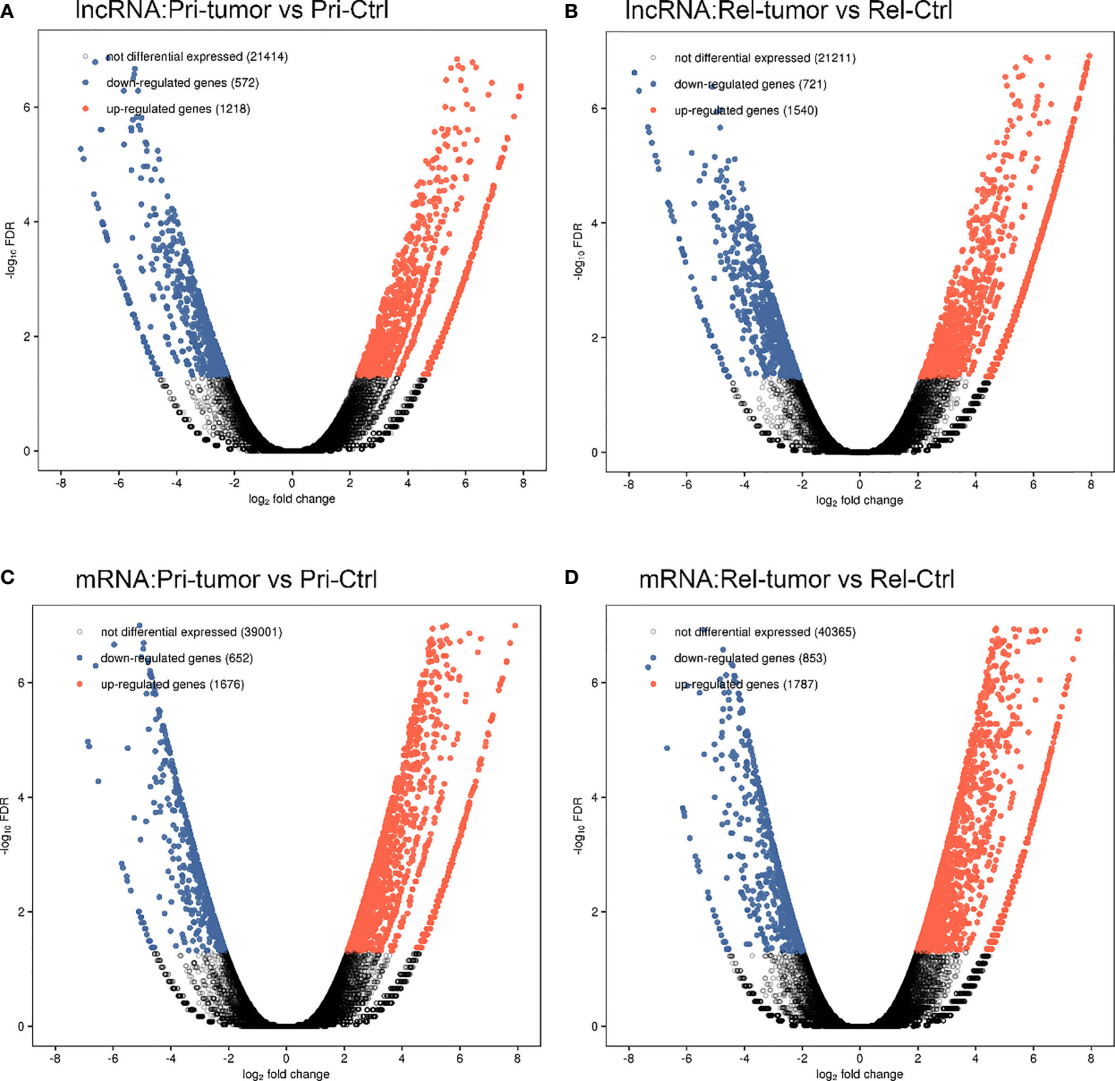
A comprehensive catalog of lncRNA genes in primary tumor, relapse tumor, benign adjacent tissue. **(A)** Volcano plot of the lncRNAs in primary tumor and benign adjacent tissue. **(B)** Volcano plot of the lncRNAs in relapse tumor and benign adjacent tissue. **(C)** Volcano plot of the mRNAs in primary tumor and benign adjacent tissue. **(D)** Volcano plot of the mRNAs in relapse tumor and benign adjacent tissue.

The overall design of the present study is shown in **Figure 1**. Employing the edge R software package, 4051 lncRNAs and 4968 mRNAs were found to be differentially expressed in the GSE101432 dataset, of which 1218 lncRNAs and 1676 mRNAs were upregulated in the primary tumor samples and 1540 lncRNAs and 1787 mRNAs were upregulated in the relapsed tumor samples (**Figure 2**).

### Identification of Differentially Expressed lncRNAs (DE lncRNAs) and mRNAs (DE mRNAs) in Relapsed HCC

**Figure 3:**
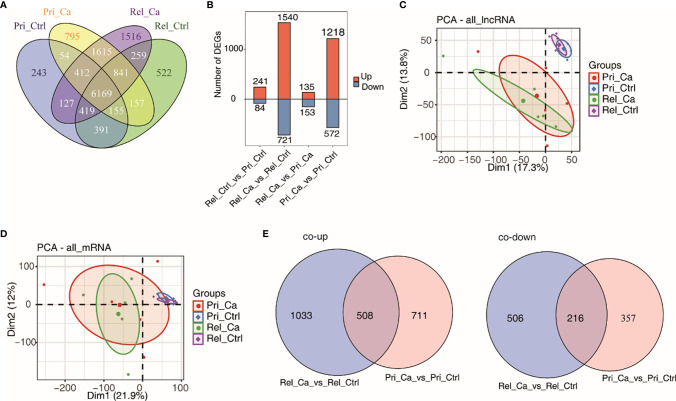
Analysis of differential expression of lncRNA and mRNA in HCC and adjacent tissues. **(A)** Venn diagram of detected lncRNA genes in primary tumor, relapse tumor, benign adjacent tissue. At least two samples with RPKM>=0.2 was considered to be detected in the group. **(B)** The number of differentially expressed (DE) lncRNAs among different groups. Bar plot showing the number of up-regulated and down-regulated DE lncRNAs. **(C, D)** Principal component analysis (PCA) of primary tumor, relapse tumor, benign adjacent tissue based on normalized lncRNAs **(C)** and mRNAs **(D)** expression level. The samples were grouped by disease state and the ellipse for each group is the confidence ellipse. **(E)** Venn diagram of detected co-up-regulated (left panel) and co-down-regulated (right panel) lncRNAs in primary tumor or relapse tumor compared with benign adjacent liver samples.

**Figure 3A** shows a Venn diagram that illustrates the overlap between the relapsed and primary tumors. Box plots for gene expression data were created to assess the distribution of DE lncRNAs (**Figure 3B**). The analysis of the data revealed that a higher number of lncRNAs in the two groups was upregulated (68%). Next, PCA was performed to compare the DE lncRNAs and DE mRNAs between the primary tumor and relapsed tumor groups (**Figures 3C, D**). It could be clearly seen that lncRNA expression differed between the primary and the relapsed tumor groups. Therefore, lncRNA might have important research potential for relapsed HCC. In addition, a Venn diagram was generated to identify the genes that were similar between all groups. The intersection of combined co-upregulated and co-downregulated lncRNAs was identified between primary tumors and relapsed tumors (co-upregulated = 508, co-downregulated = 216; **Figure 3E**).

### Co-Expression Network and Gene Enrichment Analysis

**Figure 4:**
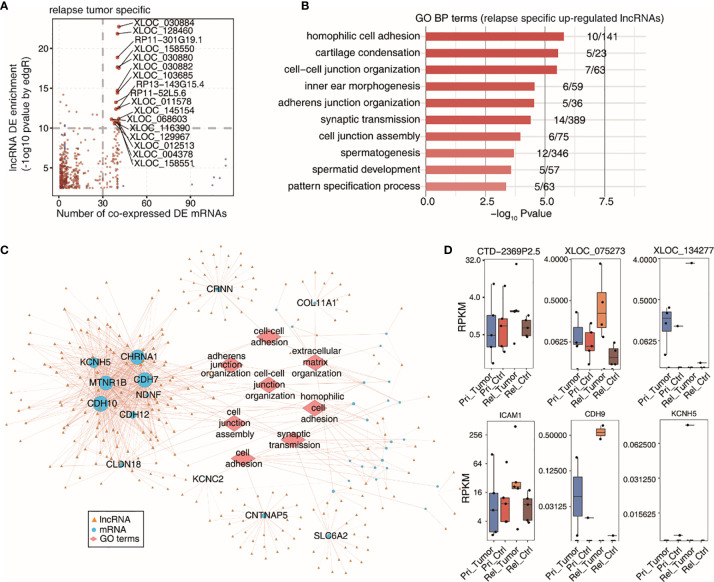
Co-expression network illustration between DElncRNAs and DEmRNAs. **(A)** Scatter plot showing specific DE lncRNAs by relapse tumor samples and its number of co-expressed DE mRNAs. Red points denote up-regulted lncRNAs involved in co-expression pairs and blue points denote down-regulated lncRNAs. **(B)** Top 10 most enriched GO terms (biological process) by DE mRNAs co-expressed with the specific up-regulted lncRNAs in relapse tumor samples. **(C)** The co-expression network between specific up-regulated lncRNAs by relapse tumor samples and co-expressed DE mRNAs which are involved in terms related to cancer metastasis. LncRNAs are represented in yellow triangle and co-expressed mRNAs are represented in blue circle and the mRNA-enriched GO terms are represented in red diamond. **(D)** Boxplots showing expression status of 3 DE lncRNAs and co-expressed mRNAs which were specific up-regulated in relapsed tumor.

We used scatter plots to determine the co-expression of DE lncRNAs with DE mRNAs in relapsed tumor samples (**Figure 4A**). The top 10 GO terms of the DE mRNAs co-expressed with the specific upregulated lncRNAs were identified using the KOBAS 2.0 server (**Figure 4B**). These processes included homophilic cell adhesion, cartilage condensation, and cell-cell junction organization. We then constructed a co-expression network between specific upregulated lncRNAs and co-expressed DE mRNAs, involving the top 10 GO terms (**Figure 4C**). We obtained the top three DE lncRNAs and co-expressed mRNAs that were specifically upregulated in relapsed tumor and that were expressed in the primary and relapsed tumors and benign adjacent tissue (**Figure 4D**).

### Identification of WGCNA Modules

**Figure 5:**
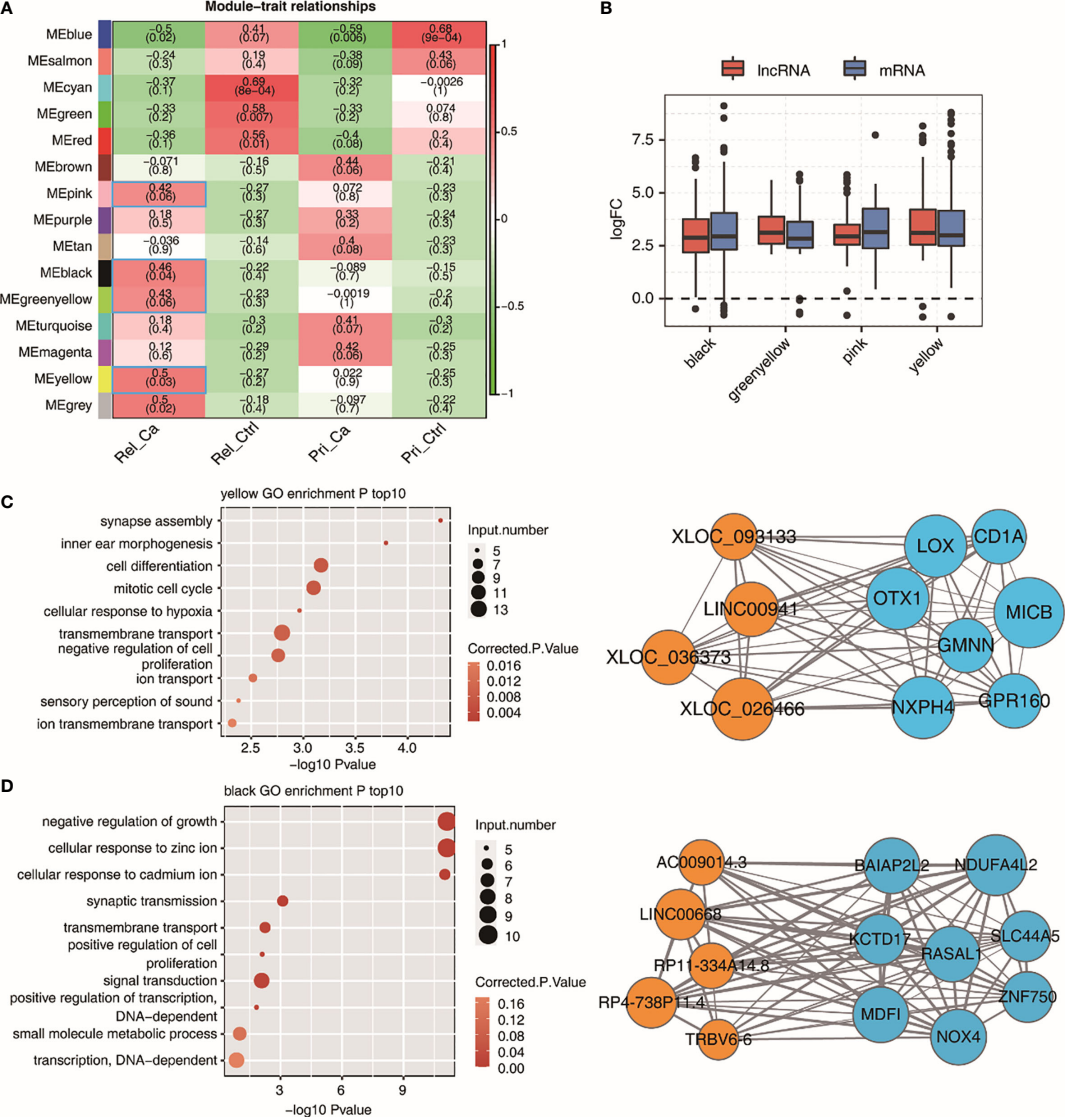
WGCNA analysis of all expressed lncRNAs and mRNAs. **(A)** Module-trait associations as computed by an LME model with all factors on the x axis used as covariates. All Pearson’s correlation value and *P* values are displayed. Module-traits associations with R and correlation>0.4 were framed with blue box. **(B)** Boxplot showing expression fold change of mRNAs and lncRNAs from the four relapse associated modules. **(C, D)** Module plots displaying the top hub genes along with the GO terms enrichment of each of the three modules, MEyellow **(C)**, MEblack **(D)** Orange circles indicate lncRNAs and lightblue circles indicate mRNAs.

We performed WGCNA on all DE lncRNAs and DE mRNAs to obtain module-trait associations, leading to the identification of 15 gene co-expression modules. (**Figure 5A**). Here, based on the R value, we chose four modules, namely black, green-yellow, pink, and yellow, and performed GO enrichment analysis on them. When comparing the fold-change in lncRNAs and mRNAs with that in the control group in the four modules that were positively correlated in relapsed HCC, all genes were found to be upregulated in the four modules (**Figure 5B**). It is worth noting that there was no statistical difference between lncRNAs and mRNAs in these four modules. The target gene analysis of lncRNAs showed that there was a high correlation between lncRNA and mRNA, which is consistent with the results of previous studies, indicating that lncRNAs might be functionally related to their neighboring mRNAs. Thereafter, we performed GO functional enrichment analysis on mRNAs co-expressed with lncRNAs in the four modules. The first 15 GO terms in the yellow and black modules were related to cell proliferation, differentiation, and survival, as well as some transcription-related biological processes; this indicates that there are multiple abnormal cell activities in relapsed HCC. Therefore, the yellow and black modules were selected for further analysis. We generated an integrated lncRNA–mRNA regulatory network based on the hub genes identified within the yellow- and black-module hub genes (**Figures 5C, D**).

### Identification of Hub lncRNAs Based on Key Modules

**Figure 6:**
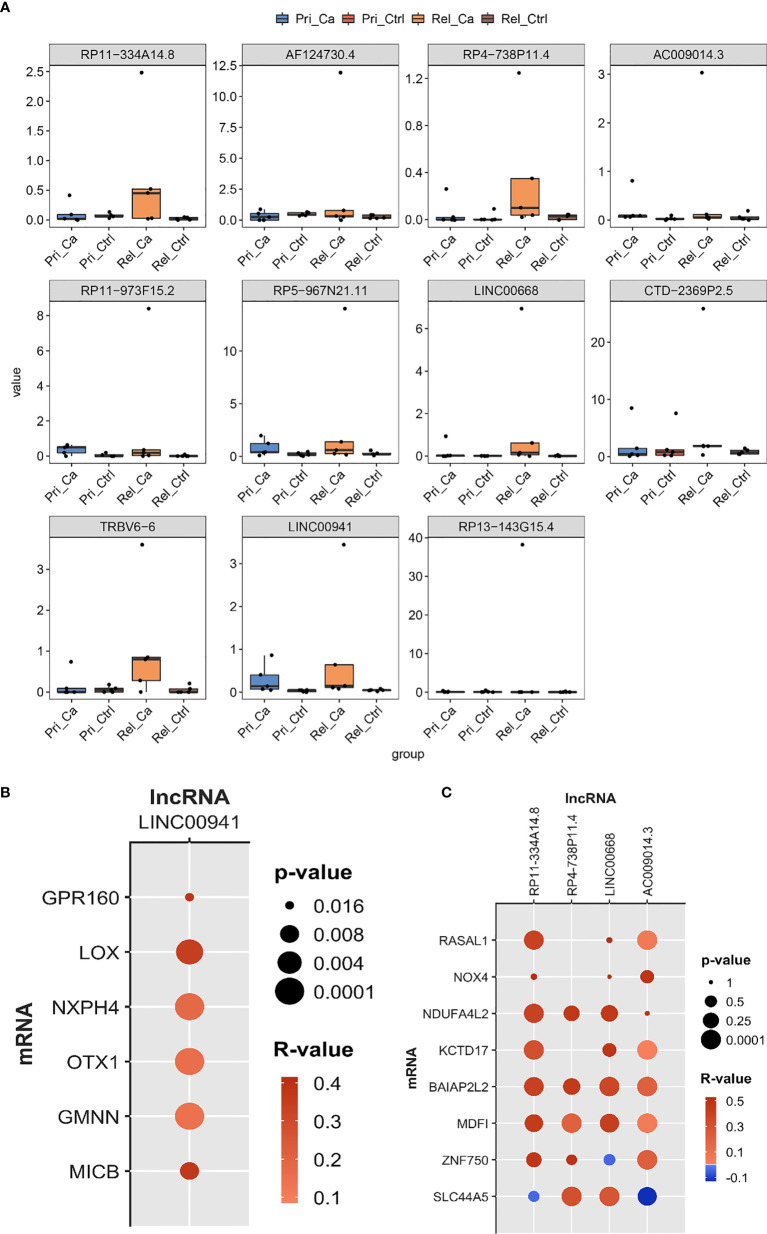
Identify hub lncRNAs based on expression level and correlation analysis. **(A)** The results of co-expression network and WGCNA selected the expression level of lncRNA in GSE101432. **(B, C)** The bubble chart was used to show the correlation analysis between lncRNAs and co-expressed mRNA.

Using the results of the co-expression network and WGCNA, we constructed a bar graph of the expression level of selected lncRNAs from the GSE101432 dataset (**Figure 6A**). According to the bar graph, we found that RP11-334A14.8, RP4-738P11.4, TRBV6-6, LINC00668, and LINC00941 expression levels were higher in the relapsed tumor group than in the primary tumor group. Unfortunately, there is a lack of relevant information in TCGA or PubMed for TRBV6-6, and thus, we abandoned the next step of research on TRBV6-6. As such, we selected RP11-334A14.8, RP4-738P11.4, LINC00668, and LINC00941, which showed higher expression in the relapsed tumor group than in the primary tumor group for qRT-PCR validation. Taking into account TCGA database correlation analysis and lncRNA qRT-PCR results, mRNA was selected for clinical sample verification (**Figure 6B**). The correlation analysis showed that LOX, NXPH4, OTX1, and GMNN were correlated with LINC00941. Further, RP11-334A14.8 was correlated with MDFI, NDUFA4L2, and BAIAP2L2, RP4-738P11.4 was correlated with SLC44A5, and LINC00668 was correlated with SLC44A5, MDFI, NDUFA4L2, and BAIAP2L2. For AC009014.3, no mRNA met the selection criteria (**Figure 6C**).

### Validation of lncRNA and Regulated Gene Expression Network in Clinical Samples

**Figure 7:**
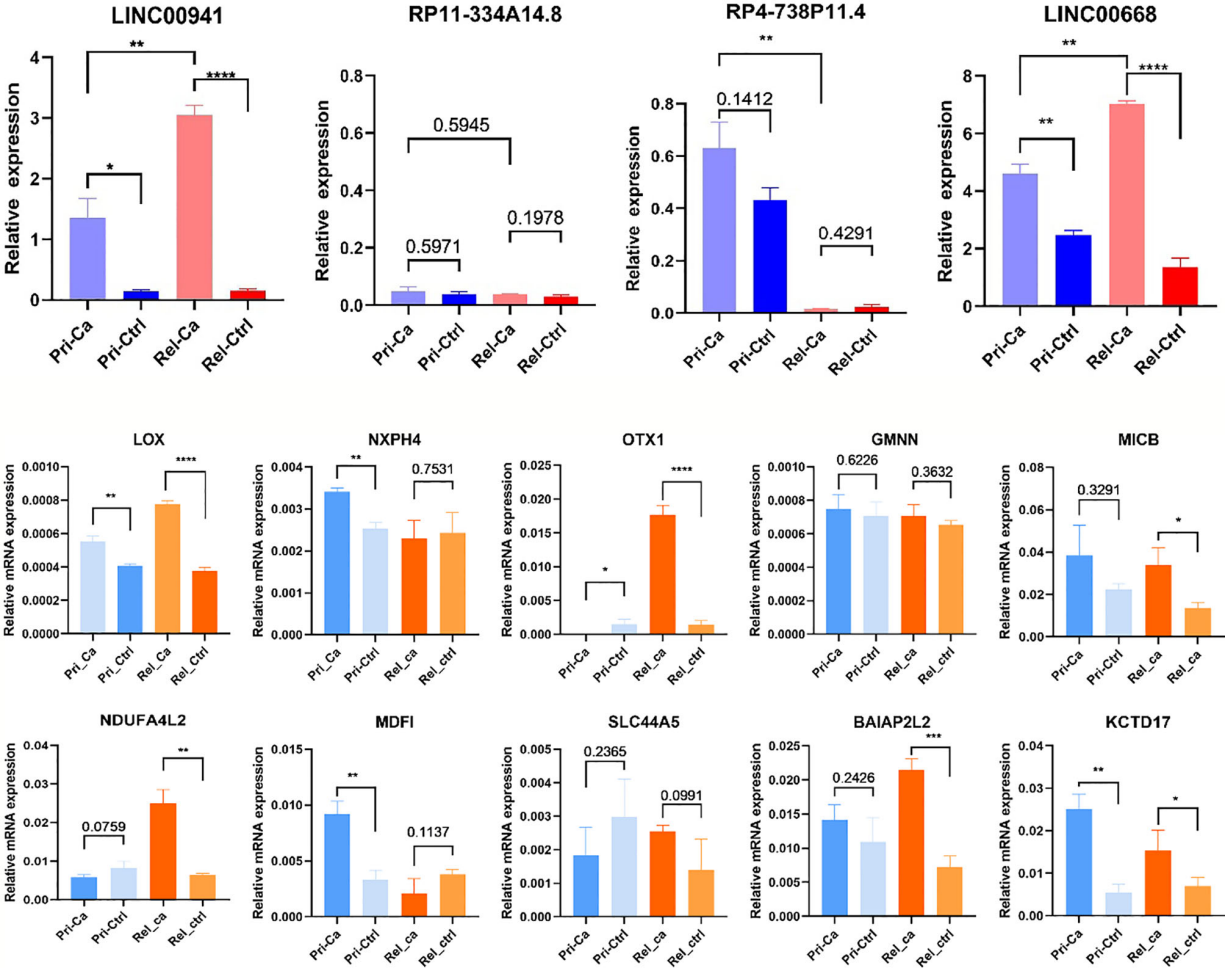
Relative expression levels of lncRNAs and mRNAs in clinical samples. (*P < 0.05, **P < 0.01, ***P < 0.001, ****P < 0.0001).

To directly validate a subset of these bioinformatics results, we assessed lncRNA and mRNA expression levels in a distinct cohort of the primary HCC, relapsed HCC, and matched normal tissue samples *via* qRT-PCR (**Figure 7**). Through qRT-PCR, we found that the expression levels of LINC00941 and LINC00668 in relapsed HCC were higher than those in primary HCC (*P* < 0.05). The expression level of RP11-334A14.8 did not change significantly in primary HCC *versus* relapsed HCC. However, RP4-738P11.4 was highly expressed in primary HCC *versus* relapsed HCC, but its expression was lower in relapsed HCC but without statistical significance; accordingly, to explain this result, other reasons for interference were considered. In addition, expression levels of LOX, OTX1, MICB, NDUFA4L2, BAIAP2L2, and KCTD17 were changed in relapsed HCC compared to those in primary HCC. Therefore, based on the results of WGCNA, correlation analysis, and qRT-PCR, we considered that BAIAP2L2, KCTD17, and NDUF4AL2 might be the mRNAs that are co-expressed with LINC00668. Meanwhile, OTX1, LOX, and MICB could be the mRNAs that are co-expressed with LINC00941.

### Expression Levels of the Two Groups of Genes in HCC Patients Across Different Tumor Node Metastasis (TNM) Stages and Tumor Grades

**Figure 8:**
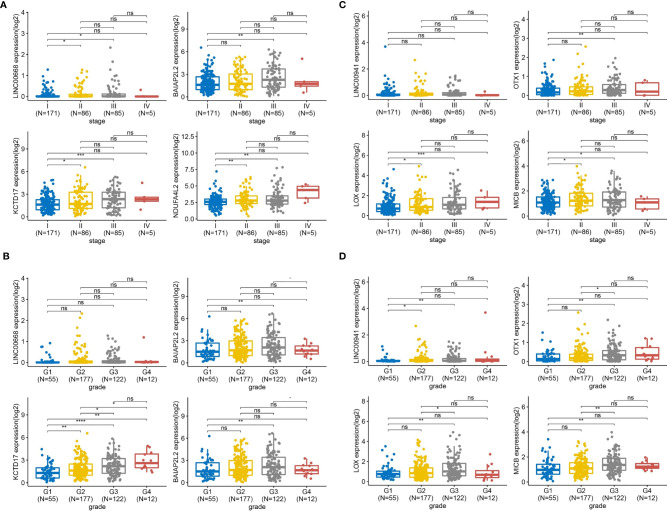
The expression levels of the two group genes in HCC patients across different TNM stages and tumor grade. **(A)** TNM stages analysis based on the expression level of LINC00668 and its co-expression mRNA in 347 HCC patients from TCGA. **(B)** Tumor grade analysis based on the expression level of LINC00668 and its co-expression mRNA in 366 HCC patients from TCGA. **(C)** TNM stages analysis based on the expression level of LINC00941 and its co-expression mRNA in 347 HCC patients from TCGA. **(D)** Tumor grade analysis based on the expression level of LINC00941 and its co-expression mRNA in 366 HCC patients from TCGA. (*P < 0.05, **P < 0.01, ***P < 0.001, ****P < 0.0001; NS, not significant).

TNM is a tumor clinical stage system. T, N, M, respectively represent the size of the primary tumor, the status of regional lymphatic metastasis, and the presence or absence of distant metastasis. Tumor grade is determined according to the degree of tumor tissue, including the degree of differentiation, arrangement, number of nuclear divisions, and local infiltration of cancer cells. To assess the expression level of LINC00668 and LINC00941 based on HCC of different TNM stages (n = 347) and tumor grades (n = 366), TCGA database was analyzed with R (**Figure 8**). As shown in **Figure 8A**, in TNM stage III, LINC00668 expression was significantly higher than that in stage I, and this difference was statistically significant. Regarding co-expressed mRNA, specifically BAIAP2L2, KCTD17, and NDUF4AL2, similar to the expression trend of LINC00668, statistically significant differences were found between stage I and III. Likewise, for LINC00668 and its co-expressed mRNA, with respect to tumor grade, expression levels were also higher with G3 disease than with G1, with statistical differences noted except for with LINC00668 (**Figure 8B**). LINC00941 was not significantly different based on TNM stage, but the expression level of co-expressed mRNA in stage III was significantly higher than that in stage I, and there was a statistical difference (**Figure 8C**). Further, for LINC00941 and its co-expressed mRNA, in terms of tumor grade, the expression level in G3 was significantly higher than that in G1, and all of these genes showed statistical significance (**Figure 8D**).

### Survival Analysis of lncRNAs Related to Relapsed HCC

**Figure 9:**
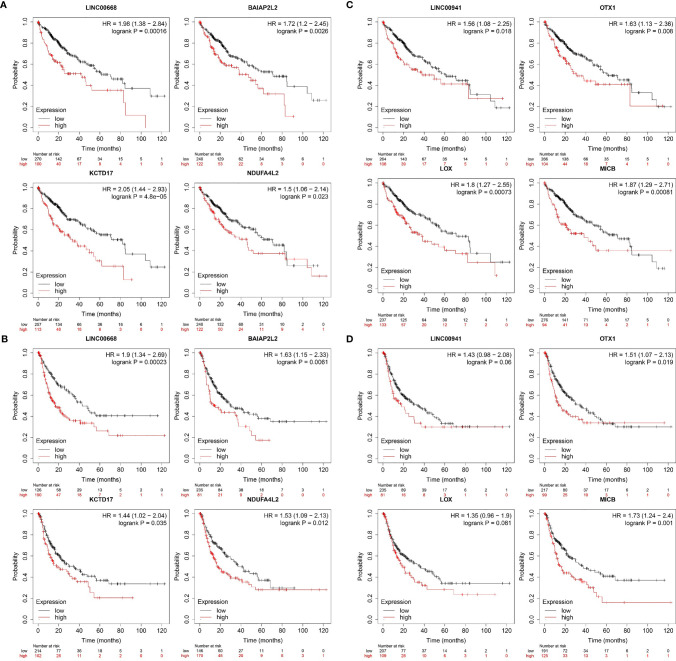
Survival analysis of hub genes in the key modules using the Kaplan-Meier Plotte **(A)** Kaplan–Meier analysis of OS based on the expression level of LINC00668 and its co-expression mRNA in 370 HCC patients from TCGA. **(B)** Kaplan–Meier analysis of RFS based on the expression level of LINC00668 and its co-expression mRNA in 316 HCC patients from TCGA. **(C)** Kaplan–Meier analysis of OS based on the expression level of LINC00941 and its co-expression mRNA in 370 HCC patients from TCGA. **(D)** Kaplan–Meier analysis of RFS based on the expression level of LINC00941 and its co-expression mRNA in 316 HCC patients from TCGA.

Kaplan–Meier plotter was used to assess the relationships between these genes and HCC patient survival, leading to the identification of prognosis-related lncRNAs and mRNA (**Figure 9**). HCC patients with high LINC00668 and LINC00941 expression had a poorer prognosis than those with low expression (*P* < 0.05). High expression of the LINC00668-co-expressed mRNAs (BAIAP2L2, KCTD17, and NDUF4AL2) was also associated with a poorer prognosis as compared to that in patients with low expression. Moreover, high expression of LINC00941-co-expressed mRNAs (OTX1, LOX, and MICB) was also associated with a poorer prognosis compared to that with low expression.

## Discussion

HCC is the second most common cause of cancer-related deaths (23). Surgical resection is the most effective first-line treatment for specific patients. However, the 5-year RFS rate after partial hepatectomy is only 48.4% (24, 25). For HCC, most studies that have published lncRNA signatures associated with prognosis have focused on primary HCC, whereas only a few have focused on relapsed HCC, and thus, finding effective biomarkers for HCC is crucial.

Data mining strategies can be used to explore significant biological phenotypes associated with high-dimensional datasets. TCGA and GEO databases, with large-scale genomic analyses, can be used to evaluate the molecular features associated with cancer outcomes. Recent developments in next-generation sequencing technologies have greatly expanded our knowledge of non-coding RNAs, and these non-coding RNAs are considerably more abundant than mRNAs (26). Many studies have revealed the role of lncRNAs in cancer development, indicating their potential as novel biomarkers for cancer diagnosis and prognosis (27–29). To better understand the molecular markers of relapsed HCC, we comprehensively analyzed the database and identified lncRNAs that can be used to predict OS and RFS. Herein, we retrieved the GSE101432 dataset containing data pertaining to HCC primary and relapsed tumors and matched non-tumor tissues from the GEO database, following which, we analyzed the differential expression patterns of lncRNA and mRNA.

LncRNA–mRNA co-expression combined with GO enrichment analysis showed that lncRNAs were related to cell adhesion, cell-cell junction organization, adherens junction organization, cell junction assembly, and pattern specification process, suggesting that lncRNAs are related to the development of cancer. In the relapsed tumor group, the co-expression network showed that upregulated lncRNA and co-expressed DE mRNA are involved in processes related to cancer metastasis; this creates conditions to promote the relapse of HCC. The CTD-2369P2.5-co-expressed protein coding gene ICAM1, which is associated with HCC, has a high level of expression, and this expression is regulated by lncRNAs. In HCC, ICR specifically affects cancer stem cell properties of ICAM-1(+) HCC cells and lncRNA ICR contributes to portal vein tumor thrombus development (30).

As a complex gene co-expression network construction method, WGCNA has unique advantages in dealing with multi-sample complex data. WGCNA technology has been leveraged to identify upregulated gene modules associated with relapsed HCC. Additionally, WGCNA has been used to analyze lncRNA and mRNA by clustering genes with similar expression patterns (31). This considers the expression of all genes evaluated in the experiment to reveal co-expressed gene clusters (modules), which are likely also co-regulated. If some genes are co-expressed in the control group but not expressed in the pathological samples, it can be assumed that the regulatory mechanism has changed, which might be the cause or result of the disease. Therefore, the genes in these modules can play a role in cancer and are therefore considered potential therapeutic targets or diagnostic/prognostic biomarkers (32). In our study, the yellow and black modules were suspected to be crucial regulators of HCC relapse and were related to cell proliferation, differentiation, and survival, as well as some transcription-related biological processes.

Given that the co-expression network and WGCNA were found to be closely associated with the relapse of HCC, we next screened for hub genes showing higher expression and evaluated their correlation in relapsed tumors. We found that the expression of RP11-334A14.8, RP4-738P11.4, TRBV6-6, LINC00668, and LINC00941 was higher in the relapsed tumor group than in the primary tumor group. We then assessed the expression of these genes *via* qRT-PCR in a separate set of primary HCC patient and relapsed HCC patient clinical samples, which confirmed that LINC00668 and LINC00941 expression levels were increased in both HCC tumor groups, whereas PHACTR2 expression was reduced in these samples relative to control tissue levels. LINC00668 and LINC00941 and their co-expressed mRNA were selected using correlation analysis, with R > 0.1 and *P* < 0.05 as the selection threshold values. Accordingly, we selected BAIAP2L2, MDFI, ZNF750, and SLC44A5 as LINC00668-co-expressed mRNAs. Moreover, GPR160, LOX, NXPH4, OTX1, GMNN, and MICB were LINC00941-co-expressed mRNAs. As a result, BAIAP2L2, MDFI, LOX, OTX1, and MICB were highly expressed in both the groups. This means that LINC00941 is co-expressed with LOX, OTX1, and MICB and is related to the relapse of HCC. Furthermore, LINC00668 was found to be co-expressed with BAIA02L2, KCTD17, and NDUFA4L2, which has biological significance for HCC relapse. Combined with WGCNA and correlation analysis, we speculate that LINC00668 and LINC00941 can affect tumor grade and TNM stages. These genes are likely to have potential prognostic value for relapsed HCC. Here, our results indicated that these genes might be new prognosis gene targets for relapsed HCC. The expression levels of LINC00668 and LINC00941 in mid-stage HCC are higher than those in the early stage, and relapsed HCC is also common in middle- and late-stage postoperative patients. This might also indicate that LINC00668 and LINC00941 are potential biomarkers for HCC recurrence. Regrettably, since there were only data for five cases of TNM IV and 12 cases of tumor grade G4 in the TCGA database, the results might be biased. These results might thus be more convincing if they can be verified by prospective research in the clinic.

LINC00941 has been reported to be associated with pro-tumorigenic and pro-metastatic behaviors during tumorigenesis, such as colorectal cancer (CRC) and gastric cancer (33, 34). Wu et al. found that LINC00941 activates the TGF-β/SMAD2/3 axis in metastatic CRC, which provides new insight into the mechanism of metastatic CRC and a novel potential therapeutic target for advanced CRC. This is a potential marker for recurrent HCC. As with LINC00941-co-expressed mRNAs, the expression of OTX1 has been found to be positively correlated with nodal metastasis status (*P* = 0.009) and TNM staging (*P*= 0.001) in HCC tissues (35). Moreover, the overexpression of OTX1 promotes the HCC proliferation, migration, invasion, and tumor angiogenesis (36). MICB is highly expressed in HCC, and its expression level is significantly and negatively associated with TNM stages (37). Among patients with different stages of hepatitis, asymptomatic individuals have higher MICB serum levels, whereas liver cirrhosis patients have lower MICB serum levels (*P* < 0.0001) compared to those in other patient groups (38). The lysyl oxidase (LOX) family members are secreted copper-dependent amine oxidases, which are characterized by catalytic activity that contributes to the remodeling of the cross-linking of the structural extracellular matrix (39). Umezaki et al. found that high LOX expression is associated with EMT markers and predicts early recurrence and poor survival in patients with HCC. This is supported by our findings, which indicate that LINC00941 and co-expressed mRNA are potential biomarkers and therapeutic targets for HCC relapse.

However, another lncRNA, located at ch18p11.31, might also play a pivotal role in the relapse of HCC. In HCC, molecular mechanistic experiments indicated that LINC00668 affects cell division, cell cycle, mitotic nuclear division, and drug-metabolizing cytochrome P450 enzymes (all *P* ≤ 0.05) (40). However, the co-expressed mRNA, BAIAP2L2, was found to be highly expressed in gastric cancer tumors and its expression was significantly correlated with tumor diameter, TNM stage, and lymph node metastasis (41). KCTD17 is upregulated in the liver tissues of obese mice and nonalcoholic fatty liver disease patients; however, few studies have been published on its role in cancer (42). NDUFA4L2 can promote cell migration, invasion, proliferation, and EMT of cancer cells under hypoxic conditions (43).

In terms of survival analysis, regarding either OS or RFS, both LINC00941 and LINC00668, as well as the co-expressed mRNAs, might be representative survival markers. In conclusion, our present research developed two lncRNA signatures for predicting the prognosis of patients with relapsed HCC. Our study indicates that the LINC00941 and LIN00668 signatures could be involved in HCC tumorigenesis, prognosis, metastasis, and relapse. Our findings might thus provide new insights into the genomic basis of relapsed HCC and provide potential prognostic targets.

Whereas the results of this research are interesting and meaningful, they have some limitations. First, some lncRNAs, such as XLOC and TRBV6-6, lacked relevant information in TCGA or other databases; thus, we were unable to conduct further research on them. Second, the WCGNA method is inherently limited by the criteria used for module selection and the network rejection threshold, which might affect the final research results. Third, we focused on the relationship of the identified lncRNAs and mRNAs, thus not further assessed the combination of some lncRNAs in indicating specific features of relapsed HCC. Fourth, the exact roles and mechanisms of the identified LINC00668 and LINC00941 in the development and progression of HCC were not assessed with *in vitro*/*vivo* experiment and needed to be further studied.

## Conclusions

The study made use of the databases from TCGA and GEO and used various bioinformatic analyses to generate novel results. The differential expression patterns of lncRNA and mRNA between primary and relapsed HCC were investigated, and numerous expression differences were discovered. The known functions of these differential expressed lncRNAs and mRNAs were analyzed for functional patterns. Significantly, two lncRNA signatures (LINC00668, LINC00941) for predicting the prognosis of patients with relapsed HCC were developed. We expect that our new resource should contribute to an understanding of the importance of lncRNA-mediated regulation, because the correlation analysis of lncRNAs and mRNA expression revealed that lncRNAs are important for HCC diagnosis and prognosis.

## Data Availability Statement

Publicly available datasets were analyzed in this study. This data can be found here: Gene Expression Omnibus accession GSE101432 and TCGA (https://portal.gdc.cancer.gov/).

## Ethics Statement

This study was approved by the Ethics Committee of the First Affiliated Hospital of Kunming Medical University. We confirmed that all studies are conducted in accordance with relevant guidelines/regulations. A signed informed consent form was obtained from each patient or their family members, who were provided a detailed explanation about the study.

## Author Contributions 

HH and ZZ: Conceptualization and financial support. YF: Database mining, sample collection, preparation of original drafts and chart construction. YY, XZ: contributed equally with first author to this work. NL, BY, ML, DZ, and LL: Technical support and manuscript revision. All authors contributed to the article and approved the submitted version.

## Funding

National Natural Science Foundation of China (No. 81960123 and 81760119).

## Conflict of Interest

The authors declare that the research was conducted in the absence of any commercial or financial relationships that could be construed as a potential conflict of interest.

## Publisher’s Note

All claims expressed in this article are solely those of the authors and do not necessarily represent those of their affiliated organizations, or those of the publisher, the editors and the reviewers. Any product that may be evaluated in this article, or claim that may be made by its manufacturer, is not guaranteed or endorsed by the publisher.
